# Balancing NAD^+^ deficits with nicotinamide riboside: therapeutic possibilities and limitations

**DOI:** 10.1007/s00018-022-04499-5

**Published:** 2022-08-02

**Authors:** Angelique Cercillieux, Eleonora Ciarlo, Carles Canto

**Affiliations:** 1 Nestlé Institute of Health Sciences, Nestlé Research Ltd., EPFL Campus, Innovation Park, Building G, 1015 Lausanne, Switzerland; 2 School of Life Sciences, Ecole Polytechnique Fédérale de Lausanne (EPFL), 1015 Lausanne, Switzerland

**Keywords:** NAD^+^, Nicotinamide, Nicotinamide riboside, Metabolic disease, Vitamin B3

## Abstract

Alterations in cellular nicotinamide adenine dinucleotide (NAD^+^) levels have been observed in multiple lifestyle and age-related medical conditions. This has led to the hypothesis that dietary supplementation with NAD^+^ precursors, or vitamin B3s, could exert health benefits. Among the different molecules that can act as NAD^+^ precursors, Nicotinamide Riboside (NR) has gained most attention due to its success in alleviating and treating disease conditions at the pre-clinical level. However, the clinical outcomes for NR supplementation strategies have not yet met the expectations generated in mouse models. In this review we aim to provide a comprehensive view on NAD^+^ biology, what causes NAD^+^ deficits and the journey of NR from its discovery to its clinical development. We also discuss what are the current limitations in NR-based therapies and potential ways to overcome them. Overall, this review will not only provide tools to understand NAD^+^ biology and assess its changes in disease situations, but also to decide which NAD^+^ precursor could have the best therapeutic potential.

## Introduction

### Sour skin

**Fig. 1:**
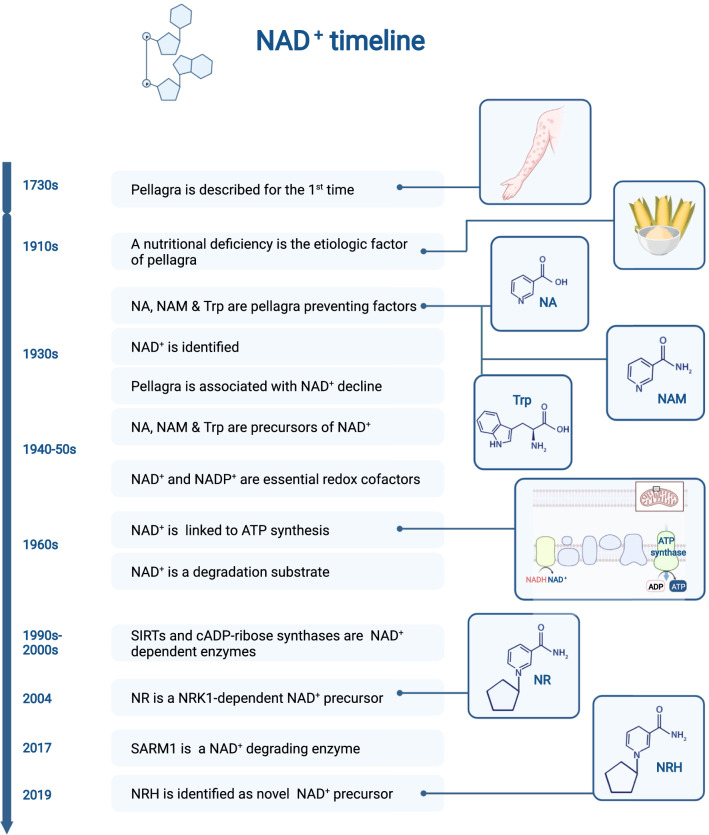
Historical timeline of some relevant discoveries in the NAD^+^ field. The story of NAD^+^ related diseases dates back to the 1700s, from the initial description of the symptoms of pellagra to the recent identification of NAD^+^ consuming activities and NAD^+^ precursors. The graph depicts a non-exhaustive list of some key milestones in the NAD^+^ field

In 1735, Gaspar Casal (1681–1759) spotted a bizarre malady in poor peasants from the region of Oviedo (Spain). These people displayed dermatitis lesions, generally manifested as rashes in exposed skin areas, such as the neck, hands, and feet. In his work, published posthumously, Casal named this disease “mal de la rosa”, due to the reddish color of the rashes, even if it was also popularly known as Asturian leprosy. Similar skin rashes were observed in peasants from southern regions of France and in northern Italy, which Francesco Frapolli referred to as “vulgo pelagrain” and used for the first time the term pellagra (“pelle” for skin and “agra” for sour, in Italian) in 1771 (Fig. 1).

The origin of this disease was not known. Multiple causes were invoked, including atmospheric influences, infectious agents, and hereditary components. Notably, patients were generally poor, subsisted on maize diets and seldomly had access to fresh meat or eggs. This led French physician Théofile Roussel (1816–1903), who became involved in politics during the Third French Republic, to legislate towards decreased cultivation of corn, substitute it with other cereal crops and increase animal husbandry [1]. Interestingly, providing French peasants with wheat instead of corn bread curtailed pellagra in France [1]. This, together with initial observations of Casal suggesting that provision of milk, cheese, and other foods would help eliminate “mal de la rosa”, became critical lines of evidence suggesting that pellagra could be a dietary problem related with maize-based diets. Therefore, the leading hypothesis at the time was that a toxic element in maize was the cause of the disease.

### The pellagra-causing factor

The scientific undermining of the causes of pellagra remained largely obscure until the early twentieth century, when pellagra reached epidemic proportions in several regions across the American continent. By 1915, Joseph Goldberger had performed a series of careful experiments in human patients unequivocally demonstrating that pellagra was not due to a toxic element in maize, but to a nutritional deficiency in maize-based diets [2, 3]. Goldberger called this nutritional factor the Pellagra preventing factor (PPF). It still took two decades to pinpoint the molecular nature of the PPF. This came from the hands of Conrad Elvehjem (1901–1962), who used the knowledge built by Goldberger to artificially induce pellagra in model organisms and identify potential palliative agents. By means of successive solvent extractions, Elvehjem and colleagues converted 400 g of liver to 2.5 g of a powder that could cure diet-induced pellagra in chickens and the canine black tongue disease, a pellagra equivalent, in dogs [4]. The upscaling and refinement of the method, now starting with several kilograms of liver, allowed the generation of crystals that led to the final identification of nicotinic acid (NA) and nicotinic acid amide (or nicotinamide, NAM) as the PPF proposed by Goldberger [4]. Proving this point, either NA or NAM treatment prevented black tongue-associated growth retardation in dogs. Only a year later, it was demonstrated that NA could also cure pellagra in humans [5]. From here on, NA and NAM were collectively referred to as niacin or Vitamin B3.

So, why did maize-based diets led to pellagra? The reason is that niacin in cereal grains is present as niacytin, which is niacin bound in a complex with hemicellulose, making niacin nutritionally unavailable. Proving this concept, the preparation method of nixtamalization, where corn is soaked and cooked in an alkaline solution, made niacin nutritionally available and reduced the chance of developing pellagra [6].

The story of pellagra became even more exciting when foods poor in NA or NAM, but rich animal proteins, were also shown to revert pellagra [7]. This led to the understanding that there might be additional PPFs. In particular, these studies led to the identification of tryptophan (Trp) as another PPF [7]. The authors noted that Trp was far less efficient than NA in preventing pellagra-associated growth retardation in rats. More specifically, Trp needed to be used at 50-fold higher concentrations than NA to achieve similar effects. The mechanism for the apparent therapeutic interchangeability of Trp and niacin, however, remained obscure. In other words, how could these structurally unrelated compounds lead to the same health benefit?

## NAD^+^ and its decline in disease

Early in the nineteenth century, a British biochemist, Arthur Harden (1865–1940), identified a yeast extract that boosted alcoholic fermentation, which he named coferment or cozymase. Hans von Euler Chelpin (1873–1964) later characterized the molecular nature of the critical factor in this extract as a nucleotide sugar phosphate. Otto Warburg (1883–1970) culminated this chain of discoveries by characterizing the molecule as nicotinamide adenine dinucleotide (NAD^+^) [6].

**Fig. 2:**
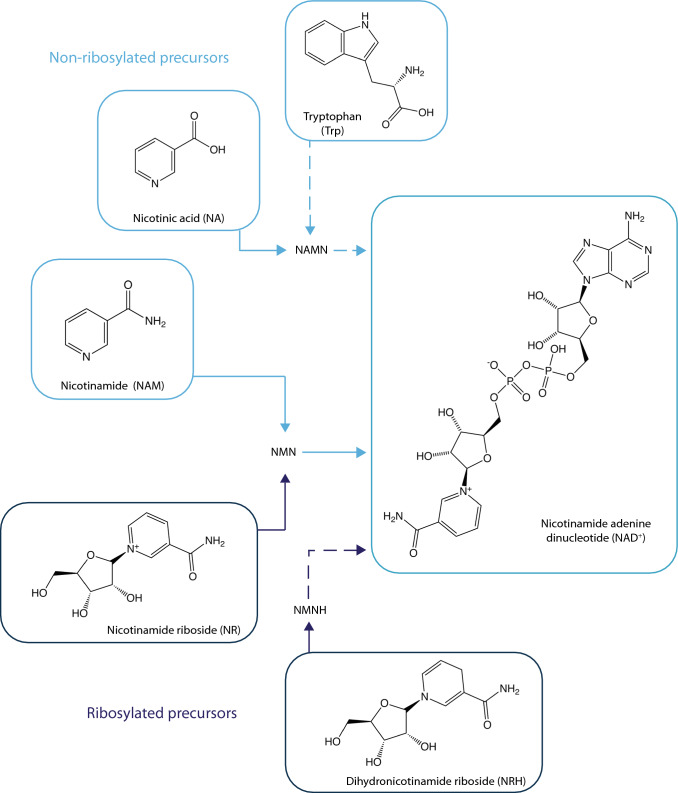
The main NAD^+^ synthesis precursors. This picture depicts the three main NAD^+^ precursors requiring a phosphoribosyltransferase reaction in their path to NAD^+^ synthesis (Tryptophan, nicotinic acid and nicotinamide) and the two main paths driven by ribosylated precursors (nicotinamide riboside and dihydronicotinamide riboside). The intermediate nucleotide forms in each path are also indicated (*NAMN* nicotinic acid mononucleotide, *NMN* nicotinamide mononucleotide, *NMNH* dihydronicotinamide mononucleotide)

In a very agile turn, Elvehjem quickly realized that NAD^+^ is a NAM containing nucleotide and that this could provide clues to understand the activity of NA and NAM on pellagra. His work demonstrated that pellagra is characterized by a sharp decline in NAD^+^ (named coenzyme I at the time) and that NA administration was able to rebuild NAD^+^ levels while curing pellagra in pigs and dogs [8]. This constituted the first evidence that NA and NAM could serve as molecular precursors for the synthesis of NAD^+^ (Fig. 2). Further solidifying the role of NAD^+^ in the pathophysiology of pellagra, Trp can also act as a NAD^+^ precursor [9], hence explaining why Trp had anti-pellagraergic effects.

### Enter NAD^+^ (I): a redox cofactor

So, what is NAD^+^ and what is it needed for? The pioneering work by Otto Warburg and co-workers in the 1930s discovered a key role for NAD^+^, and its phosphorylated counterpart NADP^+^, in hydrogen transfer biochemical reactions. The following decades unveiled how NAD^+^ and NADP^+^ are vital cofactors for most cellular oxidation/reduction reactions, where they can be reduced to NADH and NADPH, respectively, or vice versa. The NAD^+^/NADH couple primarily drives oxidation reactions, while the NADP^+^/NADPH couple drives reductive reactions (for extensive reviews on the topic, see [10, 11]). The redox potential and relative amount of the phosphorylated and non-phosphorylated nicotinamide adenine dinucleotides is very different. For instance, NAD(H) levels in liver are twofold those of NADP(H), while in muscle they are 12-fold [11]. In addition to differences between tissues, NAD(H) and NADP(H) content is highly compartmentalized in the cell, with the mitochondria harboring the higher amounts [11]. Therefore, tissues with high mitochondrial content, such as heart or kidney, display the higher NAD(H) and NADP(H) contents. Even if cellular membranes are generally impermeable to NAM-based (di)nucleotides, the mitochondrial and cytosolic pools of NAD^+^ related nucleotides and their redox states are not fully independent. Instead, they are interconnected by an intricate net of molecular redox shuttles and the recently identified mitochondrial NAD^+^ transporter [10, 12–15].

As redox cofactors, NAD(H) and NADP(H) participate in the most critical paths in cellular metabolism and mitochondrial oxidative phosphorylation. Most notably, during glycolysis, glucose can generate two molecules of glyceraldehyde-3-phosphate (G3P). This is followed by the reduction of NAD^+^ to NADH in the glyceraldehyde 3-phosphate dehydrogenase (GAPDH) reaction. Thus, glycolysis will finally render two NADH and two pyruvate molecules that can either be transformed into to NAD^+^ and lactate by the lactate dehydrogenase (LDH) reaction or transferred into the mitochondria. In the second case, the reducing equivalent of NADH is transported into the mitochondria via either the malate-aspartate shuttle or the glycerol-3-phosphate shuttle, while pyruvate has a dedicated transporter [10]. Once in the mitochondria, the pyruvate dehydrogenase complex will reoxidize NADH into NAD^+^. The mitochondrial tricarboxylic acid cycle (TCA) is a major location for the reduction of NAD^+^ into NADH molecules. Mitochondrial NADH can be re-oxidized to NAD^+^ by Complex I of the mitochondrial electron transport chain. The subsequent two electrons gained by Complex I will then be an initial step to generate a proton gradient that provides the chemiosmotic force to drive the oxidative phosphorylation of ADP to ATP, catalyzed by the F0F1-ATP synthase enzyme [10]. These processes highlight the intimate link between NAD^+^ and cellular ATP synthesis.

### Enter NAD^+^ (II): a breakdown molecule

While described in the 1960s, the role of NAD^+^ as a degradation substrate has gained attention since the early 2000s. Three major families of enzymes can cleave NAD^+^ in mammals: sirtuins, ADP-ribose transferase (ARTD) enzymes, also known as (poly)ADP-ribose polymerases (PARPs) and cyclic ADP-ribose (cADPr) synthases. In the reactions catalyzed by these enzymes, the NAD^+^ molecule is cleaved at the N-glycosydic bond, rendering ADP-ribose (ADPr) and NAM. The ADPr moiety can then be coupled to the de-ac(et)ylation of lysine residues (in the case of most sirtuins), ADP-ribosylation of proteins (some sirtuins and PARPs) or to the generation of cADPr (CD38, CD157, SARM1). These NAD^+^-cleaving enzymes are furthermore of medical relevance because they not only are indirectly affecting NAD^+^ bioavailability but also have major downstream effects on energy metabolism, DNA repair, cell survival, and senescence. In this review, we will briefly explain the cellular roles of these families of enzymes. For further insights, the reader is kindly referred to more specific reviews [16–18].

#### Sirtuins

Mammalian organisms generally express seven sirtuin enzymes (SIRT1-SIRT7). The sirtuin family of enzymes is characterized by a characteristic and evolutionarily conserved catalytic site, comprised of 275 amino acids [19]. Sirtuins are present in most cellular compartments. For example, three sirtuins are found in the mitochondria (SIRT3-SIRT5), while SIRT6 and SIRT7 are predominantly located in the nucleus [20]. SIRT2 is found in the cytoplasm [20], while SIRT1 can be found in the nuclear and cytosolic compartment and can be shuttled between them [21]. Sirtuins were initially characterized as deacetylase enzymes, but they can also act on longer acyl groups. The strength of their deacylase activity and their preferred carbon chain lengths can also differ among the 7 family members [22]. SIRT4, SIRT6 and SIRT7 can also act as mono-ADP-ribosyltransferases [23, 24].

Sirtuins have been shown to regulate enzymatic and transcriptional activities related to environmental nutrient availability. SIRT1 acts as a cofactor for multiple transcriptional regulators of mitochondrial and fatty-acid oxidation-related genes [16], while SIRT3 can directly deacetylate and control the activity of mitochondrial proteins [25]. Genetic mouse models overexpressing SIRT1 showed an effective prevention against metabolic and age-related complications, including insulin resistance, obesity and hepatic steatosis [26–28], even if overall mouse lifespan was not affected [29, 30]. In contrast, SIRT6 overexpression in mice has been shown to increase lifespan [30, 31]. Oppositely, loss of function models for SIRT1, SIRT3, SIRT6 and SIRT7 have been linked to a higher susceptibility to metabolic diseases or reduced lifespan [32–35]. SIRT2- and SIRT5-deficient mice, however, do not display overt metabolic alterations in the basal state [36, 37], while SIRT4 deficiency, in contrast to most sirtuins, enhances oxidative metabolism [38].

Given their relatively high Michaelis constant (Km) for NAD^+^, sirtuins were proposed to act as intracellular NAD^+^ sensors. The estimated total intracellular content of NAD^+^ in mammals ranges from ~ 200 to ~ 500 μM [10]. SIRT1, SIRT3 and SIRT5 show a Km of 96, 880 and 980 µM, respectively [33, 39, 40], which fits with a potential rate limitation by NAD^+^ levels, as free NAD^+^ levels in the nucleus have been estimated around 100 µM, while mitochondrial NAD^+^ levels would be around 250 µM [41]. Other sirtuins, such as SIRT4 or SIRT6, have Km of 35 and 26 µM, respectively [38, 42], which suggests that their activity is unlikely to be rate-limited by NAD^+^.

#### ADP-ribose transferases (ARTDs, also known as PARPs)

ADP-ribosylation was the first NAD^+^-dependent post-translational modification identified [43]. ADP-ribosylation, either as mono- or poly-ADP-ribosylation (MARylation and PARylation, respectively) is an ancient and evolutionarily conserved biochemical reaction predominantly catalyzed by ADP-ribosyl transferases (ARTDs, or PARPs), even if some sirtuins can also MARylate proteins [6]. Protein (poly-)ADP-ribosylation (PAR) plays roles in a large spectrum of cellular functions, including the regulation of chromatin structure, DNA repair, RNA transcription, intracellular transportation, energy metabolism, cell differentiation and proliferation, as well as determining cell death [44–46]. Part of these functions are consequent to the fact that PAR has profound effects on the biochemical properties of proteins. Most notably, PAR can serve as a binding surface for proteins harboring specific domains motifs, including PAR-binding motifs (PBMs), macrodomains, PAR-binding zinc finger (PBZ) modules, and WWE domains [46].

PARPs constitute a superfamily of up to 17 members sharing a highly conserved catalytic motif [47]. Among all these members, PARP1 and PARP2 have been the most widely studied so far because they account for most PARP activity in the cell [48]. PARP1 and PARP2 are ubiquitously expressed in mammalian tissues, with predominant nuclear localization [17]. Their activities are triggered allosterically through the binding to several nuclear proteins and to a wide range of DNA or chromatin lesions [17]. While historically described as key DNA damage repair enzymes, the generation of PARP1 or PARP2 knock-out (KO) mice suggested that the activity of either enzyme is not essential for DNA maintenance in the absence of genotoxic stress [49, 50]. However, DNA repair capacity was severely impaired in PARP1 and PARP2 KO mice when exposed to DNA damaging stresses [17]. These genetic models also revealed that PARPs regulate a plethora of other cellular processes, including cell differentiation and metabolic control.

PARP1 and PARP2 account for one-third of NAD^+^ degradation under basal conditions but become the dominant consumers in the presence of overt DNA damage [51]. This has a critical influence on cellular metabolism, both through acute actions and long-term transcriptional adaptations. The high NAD^+^ consumption rate by PARP enzymes in response to genotoxic stress results in ATP depletion, which can compromise cell survival [44]. Both PARP1 and PARP2 act as transcriptional coregulators influencing the recruitment of histone acetyltransferases and corepressors into different transcriptional complexes [17]. In some cases, PARPs repress their transcriptional activity, for example by impeding correct DNA binding through direct PARylation of the transcription factors or their cofactors [17]. PARP1 can also exert noncatalytic effects on transcriptional regulation through the direct binding to transcriptional complexes [52].

Finally, an interplay between PARP enzymes and sirtuins has been proposed. PARP1 is an avid NAD^+^ consumer, while some sirtuins, such as SIRT1, have a relatively high catalytic Km for NAD^+^ [10]. Therefore, excessive NAD^+^ consumption through PARP1 may compromise SIRT1 activity by reducing NAD^+^ bioavailability. In agreement, pharmacological or genetic reductions of PARP activity can increase intracellular NAD^+^ levels and enhance SIRT1 activity [49]. Conversely, SIRT1 has been shown to reduce PARP1 activity via direct binding and deacetylation [53]. The negative correlation of PARP and SIRT1 activities is also found in physiological scenarios. PARP activity increases in tissues from obese and aged rodents, where SIRT1 activity is lower [49, 54]. These observations suggest that physiological variations in PARP activity may have a significant impact on the function of some sirtuins, such as SIRT1.

#### ADP-ribose hydrolases

The family of cADPr synthases, including CD38 and its homolog CD157, were initially described as plasma membrane antigens on thymocytes and T lymphocytes. However, these ectoenzymes have also been found in other tissues, including muscle, liver and brain [55]. Their activity cleaves NAD^+^ to form cADPr, a secondary messenger implicated in Ca^2+^ signaling [18]. Even if CD38 inhibition in cultured cells did not alter NAD^+^ consumption rates [51], mice deficient in *Cd38* show increased NAD^+^ content in tissues such as liver, muscle, brain, and heart [55, 56]. Conversely, cells overexpressing CD38 showed reductions in NAD^+^ levels [57]. Changes in the expression of CD38 have been proposed to play a major role in NAD^+^ homeostasis in situations of inflammation, aging and senescence [58].

Recently, the Sterile Alpha and Toll/Interleukin-1 Receptor (TIR) Motif Containing 1 (SARM1) protein, which has no sequence similarity with CD38, has also been shown to produce cADPr from NAD^+^ [59]. Interestingly, SARM1 has an auto-inhibitory domain that is relieved by a NAD^+^-related metabolite, nicotinamide mononucleotide (NMN) (Fig. 2). Hence, NMN accumulation can activate SARM1, elevating cellular cADPr levels while depleting NAD^+^, ultimately leading to non-apoptotic cell death [59–61]. SARM1 was known to be important in regulating axonal degeneration [62]. However, the ubiquitous presence of SARM1 in diverse cell types suggests its participation in a wider range of cellular activities.

### NAD^+^ deficiency

During the last century, pellagra has been well characterized as a disease whose symptoms include the 3 D’s: dermatitis, diarrhea and dementia. If untreated, it can lead to a 4th D: death [63]. Even if nowadays pellagra is no longer a major medical problem and can be generally counteracted through the diet, it has not been completely eradicated worldwide. Cases of pellagra are still found in areas with poor nutritional diets, including regions in South and Western Africa, India and South America [64]. Sporadic cases are reported in poor Mediterranean regions in the south of Europe and North of Africa [64]. Less than 50 years ago pellagra was still endemic in Egypt, even if in much milder forms than in the past [65]. The availability of more nutritious foods and substitution of maize by wheat have been crucial to reduce the incidence of pellagra in most world locations. However, even in western industrialized countries, where nutritional problems rarely exist, mild pellagra symptoms are occasionally observed among chronic alcoholics, patients of with malabsorption syndromes, infectious diseases, psychiatric disorders or in response to some chemotherapeutic agents [66–69].

**Fig. 3:**
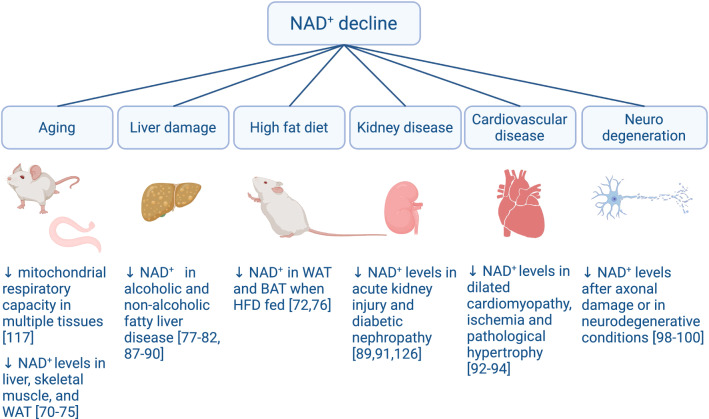
Pathophysiological situations characterized by reduced NAD^+^ levels. The figure depicts different situations were reductions of NAD^+^ levels have been reported in model organisms

Pellagra, however, is the dramatic manifestation of a severe, chronic, NAD^+^ depletion. Recent evidence in model organisms indicates that more modest decreases in NAD^+^ levels are enough to alter cellular metabolism and function and that lower intracellular NAD^+^ content is a hallmark of physiological decline (Fig. 3). Futher, geneticand pharmacologic reductions in NAD^+^ synthesis sensitize mice to the development of metabolic and neurological complications.

Aged worms display lower NAD^+^ levels, concomitant to a reduction in their mitochondrial respiratory capacity and spontaneous mobility [70, 71]. Similarly, aged mice show decreases in NAD^+^ levels in liver [72, 73], skeletal muscle [70, 73–76] and white adipose tissue (WAT) [73, 74, 77]. Dietary challenges, such as high-fat diets (HFD), have rendered conflicting and/or tissue-specific results. HFD feeding promoted a decrease in WAT and BAT NAD^+^ levels, but not in muscle [74, 78]. The situation in liver is unclear, as some studies have reported that HFD feeding compromises hepatic NAD^+^ levels [79–84], while other studies found no differences [78, 85, 86] or even increased NAD^+^ content [87, 88]. Great part of these variations might be explained by the fact that the specific diets used in these studies were different, as well as the length of the treatment and the age/strain of the animals. In contrast, other models of hepatic steatosis and hepatic damage, such as alcohol binging [89, 90] or methionine-choline deficient diets [91, 92], consistently led to a reduction in hepatic NAD^+^ levels.

Disease models have also conclusively demonstrated how functional failure is intimately connected to a decline in NAD^+^ levels in most tissues. For example, renal NAD^+^ levels are lower in situations of acute kidney injury [91] or diabetic nephropathy [93]. Similarly, reduced cardiac NAD^+^ levels are found in situations of dilated cardiomyopathy [94], ischemia [95] or pathological hypertrophy [96].

The brain constitutes a particular place of interest, as different findings in the last 20 years have built a close link between NAD^+^ biology and neurodegenerative disorders, including Alzheimer’s and Parkinson’s disease (for review, see [97, 98]). One of the main reasons for this wide impact of NAD^+^ metabolism is that NAD^+^ has a critical role in the fundamental process of axon degeneration. Axonal degeneration, also known as Wallerian degeneration, is a neuronal self-destruction program that underlies axon loss during injury and disease [99]. Three key findings related axonal degeneration to NAD^+^ metabolism. First, the fact that NAD^+^ levels decline in neurons after axonal damage or in neurodegenerative conditions [100–102]. Second, the identification of a mouse model with a spontaneous mutation that delayed Wallerian degeneration [103]. This mouse model, called the Wallerian degeneration slow (wld^s^) mice, carries a mutation that results in the overexpression of a chimeric protein (Wld^s^) composed of the ubiquitin assembly protein Ufd2a and the NAD^+^ biosynthetic enzyme Nicotinamide Mononucleotide Adenylyltransferase 1 (NMNAT1) [104, 105]. Pioneering work from the Milbrandt lab demonstrated that increased NMNAT activity was critical for the axon-sparing activity of the Wld^s^ protein [106, 107]. Finally, during the last decade multiple works identified SARM1 as an essential executioner of axonal degeneration [62]. The TIR domain of SARM1 has enzymatic activity that breaks down NAD^+^ (NADase), which is the main driver of NAD^+^ depletion upon axonal damage [59, 108]. Together, these evidences point out that alterations in NAD^+^ metabolism critically influence the process of axonal degeneration, which underlies neurodegeneration in multiple settings, from noise-induced hearing loss [109] to cognitive decline [97].

The above observations exemplify how declines in NAD^+^ are manifested early in the development of multiple pathophysiological complications. However, several challenges in our understanding remain ahead, such as:

- Why do NAD^+^ levels decline?
- To what level, and where in the cell, should NAD^+^ levels decline in order to promote a functional impairment?
- How can we evaluate NAD^+^ metabolism in a reliable fashion for the prevention and treatment of clinical patients?

In the following sections, we will try to provide some answers to the above points.

### Why do NAD^+^ levels decline? The example of aging

From a very general standpoint, NAD^+^ levels will decline when NAD^+^ synthesis rates cannot match NAD^+^ degradation rates. However, multiple factors can play in this balance, including dietary deficiencies for NAD^+^ precursors, changes in the expression levels of enzymes that transform dietary precursors to NAD^+^, or changes in the activity of enzymes that break down NAD^+^. While deficient dietary intake of NAD^+^ precursors is clearly at the origin of pellagra, this is not the case for most diseases in western civilization, largely linked to excessive food intake or aging.

To study changes in NAD^+^ turnover, quantitative NAD^+^ flux analyses are the gold standard [51]. These assays require the use of isotope-labeled NAD^+^ precursors, mass spectrometry and quantitative modeling. The labeling strategy of NAD^+^ precursors for these analyses is critical to allow the proper analysis of downstream metabolites and, therefore, the labeled precursors need to be custom synthesized. Despite these technical complexities, quantitative flux analyses for NAD^+^ metabolism have been reported in young (3 months) vs. aged (25 month) mice. This work entrained an exquisite evaluation of 21 different tissues and revealed that NAD^+^ and NADP^+^ levels modestly declined with age, but the amplitude of this change was highly tissue dependent [110]. The tissues where NAD^+^ pools were most significantly reduced where the liver, kidney, skeletal muscle, gut, colon and adipose tissues [110]. This decrease could not be attributed to impaired NAD^+^ synthesis capacity, and, therefore, are likely consequent to increased NAD^+^ consumption rates [110]. This agrees with multiple observations indicating that PARP activity is increased in aged tissues. For example, aged worms display higher PARP activity [70, 71]. Similarly, increased PARylation is also observed in aged mouse livers and in skeletal muscle [70]. NAD^+^ availability is also curtailed by increased PARP activity in accelerated aging syndromes caused by mutations in DNA repair genes or telomerase subunits, such as the Werner syndrome [111] or the Cockayne syndrome [112]. Further supporting the concept that excessive PARP activity could be prompting age-related decline comes from experiments in worms showing how PARP inhibition enhances worm lifespan [70] and delays death in accelerated aging syndromes [111, 112].

However, PARP activation might not be the only single root for age-related NAD^+^ decline in mammalian tissues. The activity of CD38 also increases in aged mouse tissues [73]. This might be surprising, as CD38 is predominantly expressed in immune cells [113]. Recent observations have provided clues on how this occurs by demonstrating that senescent cells within aged tissues drive the polarization of tissue-resident macrophages to an M1-like state characterized by increased expression of CD38 and enhanced NAD^+^ consumption [77, 114]. Therefore, the progressive increase in tissue senescent cells could also underlie age-related NAD^+^ declines. Further supporting a role for CD38 in age-related alterations in NAD^+^, genetic or pharmacological strategies aimed to reduce CD38 activity led to increased NAD^+^ levels in aged mouse tissues [73, 114, 115].

Given the above information, one would wonder about the role of sirtuins in the increased NAD^+^ consumption associated with aging. Based on experiments on T47D cells using a sirtuin inhibitor, EX527, Liu and colleagues established that sirtuins can account for one-third of basal NAD^+^ consumption [51]. However, and in contrast to what has been described for other NAD^+^ consuming enzymes, the activity of some sirtuins might be compromised in aged tissues. Part of this could be consequent to the fact that most sirtuins are characterized by a high Km for NAD^+^ [6]. Hence, decreased NAD^+^ content might compromise sirtuin activity. In line with this, the genetic deletion of PARP1 or CD38 allows for higher SIRT1 activity in mammalian tissues [49, 55]. The degree to what the activity of other NAD^+^ consuming enzymes limits sirtuin activity might explain the controversies surrounding the role of sirtuins in longevity [116, 117]. In any case, sirtuins seem to operate oppositely to PARP-1 and CD38 in relation to mammalian healthspan. This way, while genetic SIRT1 gain of function did not increase mouse lifespan [29, 30], it reduced the susceptibility to certain types of cancer and improved healthspan profiles [29]. In contrast, SIRT6 overexpression has proven to enhance mouse lifespan, even if the mechanistic aspects for this effect are still not fully understood [30, 31]. Notably, both SIRT1 and SIRT6 overexpression led to reduced hepatic inflammation [29, 30], which might prevent the age-associated increase in CD38 activity and, therefore, alleviate NAD^+^ decline. Neither SIRT1 nor SIRT6 overexpression compromised tissue NAD^+^ levels [28, 30], suggesting that increased sirtuin activity is unlikely to explain the decreases in NAD^+^ levels observed with aging.

While this section has used aging as a paradigm, similar observations have been made in other scenarios of physiological decline. This is the case, for example, of diet-induced metabolic damage. Metabolomic measurements suggest that HFD feeding enhances NAD^+^ turnover, at least in liver [85]. This is consistent with increased PARP [49, 92] and CD38 [56] activities in tissues from HFD fed mice. Notably, chronic HFD treatment decreased the activity of some sirtuins, such as SIRT1 [118] and SIRT3 [33]. Altogether, these results highlight that the most likely hypothesis why NAD^+^ levels decline in mouse tissues upon diet-induced metabolic damage are related to increased activity of PARPs and NADases following increased DNA damage and inflammation.

However, other explanations might contribute to the decrease in NAD^+^ cellular content. For example, the intracellular distribution of NAD^+^ is uneven, with mitochondria displaying two to threefold higher free NAD^+^ concentrations than the nucleus or the cytosol [10, 41]. Conditions such as aging or obesity have been related to decreased mitochondrial content in rodent and human tissues [119]. The case of skeletal muscle is particularly important, as its mitochondrial content is largely influenced by physical activity [120]. Therefore, a sedentary lifestyle could be enough to decrease skeletal muscle mitochondrial amounts and, consequently, NAD^+^ levels, even in the absence of an overt disease state or changes in NAD^+^ consuming enzymes activities. Considering that impaired mitochondrial function is a hallmark for many pathophysiological states, the relative contribution of this factor to tissular declines in NAD^+^ levels should not be neglected.

Following up on the topic, NAD^+^ content in different cellular compartments might not behave similarly in response to stress [121, 122]. Initial evidence suggested that the mitochondrial NAD^+^ levels remain unaffected following genotoxic stress, despite of a large depletion in the nuclear and cytoplasmic NAD^+^ pools [123–125]. In line with this, NAD^+^ levels in the mitochondria of livers, muscle or kidney from old mice were not significantly decreased, even if lower NAD^+^ levels were observed at the whole tissue level [110]. Oppositely, the recent discovery of the mitochondrial NAD^+^ transporter has allowed demonstrating that it is possible to deplete mitochondrial NAD^+^ levels without reducing whole cell content [13–15]. Therefore, by analyzing total intracellular NAD^+^  levels, we might be missing fluctuations at the subcellular and local level that could have a physiological impact. Oppositely, detectable changes in NAD^+^ might occur in subcellular locations with minimal impact on the main functional outcomes analyzed. New biosensors have been developed that allow the measurement of free NAD^+^ levels in different compartments [41, 126], and should pave the way towards higher resolution analyses  of subcellular NAD^+^ alterations in disease conditions.

A final element to consider is that, beyond the enzymatic characteristics of NAD^+^ consuming enzymes, we don’t really know how much NAD^+^ must decline to impair a determinate cellular function. It has been recently reported that a ~ 50% decrease in liver NAD^+^ content in healthy and obese mice does not lead to any major alteration in hepatic mitochondrial function [88], albeit it sensitized to diet-induced liver disease [127]. In this sense, it is important to note that most reports to this date evaluate NAD^+^ metabolism through NAD^+^ measurements. These, however, are not enough to understand NAD^+^ fluxes. It is likely that, in some cases, lower NAD^+^ levels could simply reflect a lower need for NAD^+^, for example due to decreased NAD^+^ turnover. Therefore, and in addition to the above-mentioned quantitative NAD^+^ flux analyses techniques [51], LC–MS based metabolomic approaches to quantitatively and semi-quantitatively measure multiple NAD^+^ metabolites in tandem can help obtaining a more comprehensive view of cellular NAD^+^ metabolism [128–130].

### Counteracting NAD^+^ deficiency with NAD^+^ precursors: the initial steps

The above section highlights how multiple situations of physiological decline are accompanied by a reduction in NAD^+^ levels. Counteracting NAD^+^ deficitscan be done through 2 main types of strategy. The first one consists in blocking the activities of NAD^+^ consuming enzymes. While pharmacological and genetic strategies to block either PARP or CD38 activities have proven valuable to increase NAD^+^ bioavailability in rodents (see [98] for review), their clinical translation has to be closely scrutinized, as these enzymes have critical roles in DNA repair, chromosomal maintenance and immune function, among others. The second strategy consists in restoring NAD^+^ levels through the administration of NAD^+^ precursors.

Less than 20 years ago, three main NAD^+^ precursors, using independent synthesis paths, were characterized in eukaryotes (Fig. 2). NAD^+^ can be synthesized from Trp through the eight step de novo pathway [10]. While in mice Trp can eventually be a major precursor for hepatic NAD^+^ synthesis, this is not the case in humans. One of the reasons for that is that the de novo pathway has a branching point in its fifth step, which is the formation of the unstable α-amino-β-carboxymuconate-ε-semialdehyde (ACMS) [131]. ACMS can undergo spontaneous cyclization forming quinolinic acid, which subsequently serves as an NAD^+^ precursor [131]. However, this spontaneous reaction only occurs when ACMS levels overwhelm the enzymatic capacity of the ACMS decarboxylase (ACMSD), which leads to the complete oxidation of ACMS via the glutarate pathway and the tricarboxylic acid (TCA) cycle [10]. This explains why, in general, Trp is considered a rather poor NAD^+^ precursor in vivo. Interestingly, a mouse model overexpressing ACMSD was generated to better model the human de novo pathway. This rendered mice, as humans, niacin dependent to sustain their tissue NAD^+^ levels [132]. A recent strategy to try to exploit Trp as a NAD^+^ precursor consists in the inhibition of ACMSD [91]. However, its efficacy in humans is not yet demonstrated.

Niacin generically refers to two molecules, nicotinic acid (NA) and nicotinamide (NAM). NA synthesizes NAD^+^ through the shorter, 3-step, Preiss-Handler pathway [10]. In this path, the rate-limiting step occurs after the cellular uptake of NA, when the NA phosphoribosyltransferase (NAPRT) transforms NA into NA mononucleotide (NAMN). NAMN will then require 2 additional enzymatic steps to generate NAD^+^ [10]. NA has been used for decades as a treatment for dyslipidemia [133]. However, NA is rarely used in clinical practice these days due to some of its side effects. Most notably, NA induces cutaneous flushing, which compromises patient compliance [134]. NA-induced flushing is not related to NAD^+^ synthesis, but to the activation of a G-coupled receptor, GPR109A [135]. The low presence of NA in circulation suggests that the activation of this receptor is unlikely to be a native function of NA, but rather an effect from pharmacological dosing. Some evidence suggests that the ability of NA to improve lipid and cholesterol profiles is related to the activation of the GPR109A receptor, including: work on genetically engineered mouse models [136, 137], the fact that NA requires a 100-fold higher dose to treat lipidemia than to prevent pellagra, and the failure of NAM to provide similar benefits [137]. However, GRP109A is not expressed in the liver [137–139], a central hub for HDL and LDL metabolism. Also, GPR109A agonism through other molecules did not recapitulate the lipid lowering effects of NA [140]. Therefore, there are still some mechanistic aspects on the clinical actions of niacin that remain unclear. NA can act as a potent NAD^+^ precursor in liver and kidney, which are the main tissues expressing NAPRT [141]. However, NA is a poor NAD^+^ precursor beyond these tissues [137, 142]. Therefore, and despite some clinical success, the side effects of NA administration prevent its use as a dietary supplement for at-risk populations.

NAM is the third classical form of NAD^+^ precursors. Some evidence suggest that NA, as well as the NAD^+^ contained in foods, might be largely converted to NAM in the gastrointestinal tract [143]. Notably, NAM is also the end-product of cellular NAD^+^-consuming activities, hence the cell is equipped with the enzymatic machinery to recycle NAM and use it for the re-synthesis of NAD^+^. The first step in the synthesis of NAD^+^ from NAM is catalyzed by the Nicotinamide phophoribosyltransferase (NAMPT) enzyme [144, 145]. By virtue of this enzymatic reaction, NAM is transformed into nicotinamide mononucleotide (NMN), which is then used by NMNATs to synthesize NAD^+^ [10]. The fact that the genetic ablation of NAMPT leads to embryonic lethality in mice at day 10.5 suggests that NAM is probably the main precursor used to sustain tissue NAD^+^ levels in mammals [146]. However, NAM supplementation has not achieved the therapeutic success of other NAD^+^ precursors in pre-clinical and clinical settings. One potential explanation is that, unlike other NAD^+^ precursors, NAM can exert end-product inhibition on NAD^+^ consuming enzymes, which are the ones aiming to be activated through NAD^+^ synthesis [10]. Therefore, the therapeutic dosing windows for NAM in different indications might need to be scrutinized. A second complication in the use of NAM arises from the fact that NAM can be shunted away from NAD^+^ synthesis. This way, NAM can be methylated by the NAM n-methyltransferase enzyme (NNMT), generating 1-methyl-NAM (mNAM) [147]. This reaction requires using S-adenosylmethionine (SAM) as a methyl donor, which also acts as a cofactor for other cellular methylation reactions. Long term or high doses of NAM have led to detrimental effects, such as the development of a fatty liver, by reducing the availability of methyl groups [148]. Recent experiments have highlighted how, in humans, NAM promotes a larger methyl donor depletion than NA at equimolar treatments [149], further stressing the need for caution in the use of NAM as a long-term NAD^+^ boosting strategy.

Therefore, all classic NAD^+^ precursors show some shortcomings when it comes to their clinical application. Would it be possible to enhance NAD^+^  biosynthesis through other molecules that could avoid these side-effects?

## Nicotinamide riboside: from discovery to clinical application

### A new NAD^+^ precursor found in milk

In their seminal 2004 paper, Bieganowsky and Brenner identified nicotinamide riboside (NR) as a new NAD^+^ precursor in eukaryote organisms [150]. Most importantly, NR used a different, previously unknown, path for NAD^+^ synthesis [150] (Fig. 2). Later work showed how NR could increase NAD^+^ levels in cultured mammalian cells [151], rodent tissues [152] and peripheral blood mononuclear cells in humans after oral administration [153]. The ability of NR to act as a NAD^+^ precursor in mammalian cells and organisms has since been replicated by multiple independent labs (see [154] for review).

When screening for natural sources of NR, Bieganowsky and Brenner found it in a vitamin fraction of cow's milk [150]. The presence of NR in milk was later confirmed to be in the low micromolar range in different cow milk preparations [155], as well as in human milk [156]. However, when it comes to human nutrition, the main source of NR might not be milk. Indeed, NR is also a degradation product of NAD^+^ in the small intestine [143]. Initial studies of NAD^+^ hydrolysis using rat intestine brush border membranes demonstrated that NAD^+^ was fully degraded within 20 min, coinciding with the appearance of adenosine, NMN and NAM, at molar proportions of 1:0.7:0.3, respectively [157]. After the exhaustion of NAD^+^, NAM levels remained unaltered, while the amount of NMN declined and gave raise to NR, whose levels increased until the exhaustion of NMN [157]. A second study, directly perfusing the duodenum of rats with labeled NAD^+^, revealed similar findings: a quick decline in NAD^+^, resulting in an initial rise of NMN, followed by a large increase in NR, which became the main product in the intestinal content within minutes [143]. Therefore, mammalian organisms might obtain NR as a product of NAD^+^ degradation in the gut. However, the amounts of NR finally absorbed and reaching circulation are unclear, as NR can be further degraded to NAM by intestinal cells [143].

Another possible source of NR could be its release from a particular cell/tissue/organ to feed other cells/tissues/organs in a paracrine or systemic manner. Initial evidence for this possibility was obtained in yeast, where the endogenous production and release of NR could be detected in response to NA/NAM-mediated salvage [158]. It was hypothesized that the release of NR could be used as a way of communication within a yeast cell colony [158]. More recently, elegant experiments from the Nikiforov lab revealed how cultured mammalian cells grown in the presence of NA/NAM could also produce and release NR and its deamidated counterpart, Nicotinic acid Riboside (NAR) to the extracellular milieu [159]. More recently, the generation and release of NR was also observed in primary mouse hepatocytes and in mice [128]. Collectively, these results suggest that NR utilization in mammalians might not be exclusively related to the dietary supply, but to endogenous production and distribution within an organism.

### Nicotinamide riboside kinases (NRKs) and NR-induced NAD^+^ synthesis

NR is internalized into the cell by the *Nrt1* transporter in yeast [160] and by equilibrative nucleoside transporters (ENT1, ENT2 and ENT4) in mammalian cells [161–163]. Once internalized, NR is quickly phosphorylated by the nicotinamide riboside kinase enzymes (NRKs). NRKs were identified as NR kinases in yeast through a biochemical genomics approach, screening multiple pools containing different open reading frames [150]. PSI-BLAST analyses revealed that the predicted *S. cerevisiae* NRK polypeptide (Nrk1) had an orthologous human gene product (locus NP_060351) at 9q21.31, encoding a polypeptide of 199 amino acids (NRK1). Even more interestingly, a potential second human gene product (locus NP_733778) with NRK activity was identified. It was 57% identical to human NRK1, and therefore called NRK2 [150]. NRK2 is a 230 amino acid splice form of what was at that time described as a 186 amino acid muscle integrin β1 binding protein (ITGB1BP3; MIBP) encoded at 19p13.3 [164].

To establish that yeast Nrk1 was the only enzyme that phosphorylates NR, Bieganowsky and Brenner devised a brilliant strategy using *S. cerevisiae* mutants without the *QNS1* gene. Unlike mammals, NAD^+^ synthesis from NAM, NA and Trp requires the *QNS1* gene, encoding the glutamine-dependent NAD^+^ synthase [165]. Therefore, *QNS1* mutant yeast require other NAD^+^ precursors for their viability, like NR. Indeed, NR rescues the viability of the *QNS1* mutant, but could not rescue viability when a double mutant for *QNS1* and *NRK1* was generated [150]. This certified the NRK1 was the enzyme phosphorylating NR. Further, the introduction of either human NRK1 or human NRK2 were able to rescue the viability of NR-treated *QNS1 NRK1* double mutant yeast [150]. This conclusively demonstrated that NRKs account for the phosphorylation of NR and constitute a first step in its path as a NAD^+^ biosynthetic precursor.

#### Nicotinamide riboside kinase 1 (NRK1)

While NRK activity had been fuzzily described in the past, its metabolic role was unclear. Initially characterized as a kinase activity that could phosphorylate 3-deazaguanosine and tiazofurin [166], the NRK enzyme was successfully purified from human placenta by Sasiak and Saunders 25 years ago [167]. The human placental NRK appeared to be a monomer, running on SDS-PAGE with a molecular weight around 29 KDa [167]. Once NRKs were identified as the critical mediators for NR-induced NAD^+^ synthesis, structural studies quickly followed.

Human NRK1 spans 199 amino acids and consists of five-stranded parallel β-sheets, flanked by 2 α helices on one side (Helices E and A), a helix on the other (Helix B) and a lid domain composed by two additional helices (C and D) connected by a 12-amino acid loop [168, 169]. The structural conformation of NRK1 heavily resembled that of other kinases [168, 169], which facilitated the understanding of the role of the different domains. The active site is located at the top of the 5-stranded parallel β-sheets, and the lid domain assists the binding of ATP and the phospho-acceptor substrate. These structural studies revealed potential critical residues for NRK-mediated catalysis, including K16, D36, D56 and E98. Mutagenesis analyses confirmed that the D36A mutation abolished NRK1 activity in in vitro assays [169], yeast [168] and mammalian cells [163]. Other mutations, such as E98A, led to an unstable protein in mammalian systems [163]. The kinetic parameters of human NRK1 have also been well characterized, with a Km for NR and ATP in the low micromolar range [167–170]. Interestingly, NRK1 can also efficiently phosphorylate NAR, which can also act as a NAD^+^ precursor [168].

NRK1 is a cytosolic protein [162, 171]. While detectable at the mRNA level in most tissues, NRK1 is predominantly present in liver and kidney [163]. NRK1 is encoded by the *Nmrk1* gene, whose transcriptional regulation is far from understood. A study of the circadian hepatic transcriptome identified that *Nmrk1* expression is upregulated during the feeding phase [172]. High-fat feeding does not seem to alter hepatic NRK1 levels in mice [85, 86], while it increased *Nmrk1* expression in muscle [173]. An increase in NRK1 content was observed in liver samples from aged mice [86]. In humans, a cross-sectional study of 40 monozygotic twins discordant for body mass index, revealed that *Nmrk1* transcripts were significantly down-regulated in the subcutaneous adipose tissue of the heavier co-twins [174]. In addition, *Nmrk1* expression inversely correlated with adipose tissue inflammation markers [174].

Altogether, our understanding of NRK1 biology is still in its infancy. Its high expression in liver and kidney suggests that these are the tissues where alterations in NR availability would have the greatest impact. However, there are still many gaps in our understanding of the elements regulating *Nrmk1* transcription, as well as on potential post-translational or allosteric mechanisms for NRK1 regulation. Also, while NRK1 is a recognized kinase for NR and NaR, we do not know if it could also phosphorylate other endogenous nucleosides or metabolites.

#### Nicotinamide riboside kinase 2 (NRK2)

The NRK2 protein is encoded by the *Nmrk2* gene and constitutes a longer 230 amino acid (aa) splice variant of a previously described 186 aa muscle integrin binding protein (MIBP) [164]. Importantly, MIBP lacks the catalytic domain of NRK2. The expression of *Nmrk2* is largely muscle specific, albeit it can also be detected at lower levels in cardiac tissue [163, 164, 171]. Initial efforts in cultured myocytes suggested that MIBP overexpression compromised myogenesis [164]. Later, studies in zebrafish identified that Nrk2b, the zebrafish homolog of NRK2, was required for musculoskeletal development [175]. The absence of Nrk2b led to structural muscular problems, including alterations in the extracellular matrix and abnormally long and disorganized muscle fibers [175]. As Nrk2b contains an integrin binding domain, it was proposed that the Nrk2b was localized to myotendinous junctions to provide a local NAD^+^ supply, which could explain the need for a second NRK enzyme in muscle. Supporting the concept that the main function of Nrk2b is to sustain NAD^+^ levels, the phenotype of Nrk2b deficient zebrafish was rescued following administration of exogenous NAD^+^ [176]. The role of NRK2 in the maintenance of NAD^+^ levels in mammalian muscle, however, is not as clear. Experiments in primary muscle cells demonstrated that the absence of NRK1, NRK2 or both does not compromise baseline NAD^+^ levels [171]. Deletion of NRK1 or NRK2 partially impaired NR-induced NAD^+^ synthesis in muscle, which was only completely abrogated when both enzymes were knocked-out [171]. Also, the absence of NRK1, NRK2 or both enzymes did not lead to any evident abnormality in muscle morphology or function in young healthy mice ([163, 171]; Ratajczak J. and Canto C., unpublished observations).

A key difference between *Nmrk1* and *Nmrk2* expression is that the later seems to be very responsive to cellular damage and metabolic stress. This was initially illustrated by the Milbrandt lab, who reported that *Nmrk2* expression was dramatically up-regulated following injury in dorsal root ganglion neurons [177]. A striking element in this observation is that NRK2 levels are undetectable in the brain under normal conditions [163]. In muscle, *Nrmk2* expression was also largely increased in mouse models of traumatic lower limb injury [178] or severe muscle myopathy [179]. Also, increased *Nmrk2* expression levels were observed in muscle in response to high-fat feeding [173]. Sharp changes in *Nmrk2* expression can also be observed in cardiac muscle in response to stress. For example, *Nmrk2* mRNA expression increased ~ 90-fold in the heart of mouse models of lethal cardiomyopathy [180]. Similarly, *Nmrk2* expression increased several fold in mouse and human ischemic hearts [181], as well as in murine and human samples of dilated cardiomyopathy [94, 182].

The reasons why *Nmrk2* expression is so responsive to stress are not well understood. From a functional standpoint, the pathogenesis of most, but not all [183], disease models where *Nmrk2* expression increases can be improved via NR administration [94, 177, 180]. This suggests that the induction of *Nmrk2* could be a cellular adaptation to support NAD^+^ generation. Accordingly, most of these disease models display tissue NAD^+^ deficits [10, 180]. This hypothesis is very nicely supported by the observation that FK866-driven inhibition of NAMPT activity in muscle or cardiac cells is enough to trigger a decrease in NAD^+^ levels and to induce *Nmrk2* expression [94, 171]. The treatment of cells with NR was enough to prevent the effects of FK866 on NAD^+^ levels and *Nmrk2* transcription [94, 171]. Thus, it seems plausible that *Nmrk2* levels increase as a response to a situation of energy and NAD^+^ crisis, in order to maximize NR-induced NAD^+^ synthesis. An obvious question is why this response is so specific to *Nmrk2*. This might be explained by some of the specific features of the *Nmrk2* promoter. The analysis of the 5’-regulatory sequences of the *Nmrk2* gene revealed some putative peroxisome proliferator-activated receptor (PPAR) binding sites [94]. Among the different PPAR family members, only PPARα overexpression stimulated *Nmrk2* expression [94]. The activation of PPARα in the *Nmrk2* promoter upon energy stress might be consequent to the activation of the AMP-activated protein kinase (AMPK). In line with this, pharmacological activation of AMPK was enough to increase *Nmrk2* expression [94]. AMPK can lead to the direct or indirect phosphorylation of PPARα [184], which provides an elegant link between energy stress and *Nmrk2* expression.

#### Nicotinamide mononucleotide adenylyltransferases (NMNATs)

The phosphorylation by NRKs transforms NR into NMN, which is later transformed to NAD^+^ via the NMNAT enzymes. This is a common enzymatic step for all known NAD^+^ precursors to date. Here, we will only provide an overview on NMNATs biology. For more extensive information, the reader is referred to recent excellent reviews on this field [185, 186].

There are three NMNAT enzymes in mammals (NMNAT1-3), which differ in their tissue pattern expression and cellular localization. NMNAT1 is a nuclear enzyme that is ubiquitously expressed [187, 188]. In contrast, NMNAT2 is associated with the Golgi and acts in the cytoplasm [188, 189], while NMNAT3 has been identified in both cytosolic and mitochondrial compartments, with cell/tissue-specific subcellular localization patterns [189–191]. The presence of NMNAT enzymes in different compartments suggest that salvage of NAD^+^ precursors might occur in multiple cellular locations. For NR, this might not be the case, as NRK1, the most widely expressed NRK enzyme, is cytosolic [163]. While it was speculated that NMN might be transported across organelle membranes, recent work demonstrated that NAD^+^, instead of NMN, is the transported metabolite, at least in the case of mitochondrial import [12]. This suggests that NMNATs might control local NAD^+^ salvage, probably from the NAM produced by NAD^+^-degrading activities. NMNATs can also help preventing the accumulation of NMN, which has been shown to be toxic in neuronal cells [192].

The genetic deletion of individual NMNAT enzymes has demonstrated that their functions are not redundant. Knockout mouse models for NMNAT1, NMNAT2 or NMNAT3 are all lethal, yet at distinct timings in life and due to different causes [190, 193, 194]. A second argument supporting that NMNAT enzymes are not redundant and that compartment-specific NAD^+^ synthesis is critical was found in experiments using cultured dorsal root ganglion neurons. There, loss of axonal NMNAT2 results in axonal degeneration [195]. The restoration of NMNAT2 activity can prevent degeneration of the axon [195], but this was not the case when NMNAT1 was overexpressed [196]. In contrast, the overexpression of a NMNAT1 form that localized to the axonal cytoplasm, instead of the somatic nucleus, could effectively block degeneration [197]. These works on NMNATs demonstrated that the location of NAD^+^ synthesis is crucial for cellular functions. Therefore, future efforts will not solely need to analyze total intracellular NAD^+^ levels, but also subcellular localization in order to understand the influence of changes in NAD^+^ on functional physiological outcomes.

### From bench to bedside

The fact that NR did not activate GPR109A [152] and that it stimulated NAD^+^ synthesis in multiple mammalian cells [151, 152] quickly built the concept that NR could be a valuable therapeutic alternative to niacin. Early reports on mouse interventions with NR emerged in 2012 and built up a considerable body of evidence supporting the promising prospects of this molecule. This early evidence and the successful up-scaling of NR chloride synthesis to metric ton ranges created the necessary momentum to move NR into clinical settings. The dosages of NR used in most pre-clinical mouse studies generally range between 200 and 500 mg/kg/day, with no toxic side effects reported. Toxicology studies in rats revealed that NR is a very well-tolerated molecule, with side effects only observed at doses as high as 1 g/kg/day [198, 199]. Similarly, a mouse study reported how a very high NR concentration (9 g/kg of diet, approximately 1 g/kg of body weight/day) led impaired glucose tolerance and promoted white adipose tissue inflammation [200]. These toxicology profiles in rodents are very reminiscent of those of the classic NAD^+^ precursor, NAM [198, 201]. In humans, NR has been administered to doses up to 2 g/day, without any apparent side effects [153, 202–205].

In this section, we will not summarize the full spectrum of pre-clinical trials performed in using NR, but only on those performed in mammalian organisms and in the areas of metabolic disease and aging, where clinical data is also already available (see also Table 1).

**Table 1:** Pre-clinical and clinical comparisons of the effects of NR supplementation

	PRECLINICAL TRIALS (RODENTS)	CLINICAL TRIALS
SAFETY	• No toxic side-effects observed with 200–500 mg/kg/day; The lowest observed adverse effects occurred at 1 g/kg/day NR in rats [198, 199] • Approximately 1 g/kg/day of NR led to impaired glucose tolerance and WAT inflammation in mice [200] • Similar toxicology profile of NR and NAM in rodents [201]	• No adverse effects with NR administration up to 2 g/day [153, 202–205]
METABOLIC DISORDERS (I): GLUCOSE HOMEOSTASIS	• NR prevented and/or treated diet-induced glucose intolerance in some [80, 152], but not all studies [206–208] • NR improved glucose homeostasis in mice treated with a high-fat diet (HFD) and streptozotocin (STZ) [84] • NMN, which can be dephophorylated to NR, improved diet-induced glucose intolerance [74]	• NR supplementation (1–2 g/day, for 6 and 12 weeks, respectively) failed to improve insulin sensitivity or influence glucose metabolism in overweight/obese pre-diabetic patients [202, 209, 210] • NMN supplementation (250 mg/day for 10 weeks) improved insulin sensitivity in overweight/obese pre-diabetic postmenopausal women [211]
METABOLIC DISORDERS (II): HEPATIC DAMAGE	• NR reverted NAFLD; Improved glucose homeostasis in mice submitted to either HFD and STZ [84] or high-fat high-sucrose (HFHS) diets [80] • NR prevented alcohol-related liver damage [89] • NR ameliorated diet-induced hepatic fibrosis [212] • NR promoted liver regeneration after partial hepatectomy [213]	• NR in combination with glutathione precursors and L-carnitine tartrate reduced liver fat and circulating levels of ALT and AST in NAFLD patients [214] • A tendency towards a reduction in hepatic lipid content was observed when obese pre-diabetic individuals were supplemented with NR, albeit the study was not designed/powered for this endpoint [202]
METABOLIC DISORDERS (III): MUSCLE PERFORMANCE	• NR had no impact on exercise performance or muscle mitochondrial content in mice fed a regular diet [152, 215] • No alterations in the skeletal muscle acetyl-proteome after NR treatment [207] • NR prevented the decline in muscle mitochondrial function triggered by chronic high-fat feeding [152]	• Acute (2 h) or short-term (7 days) NR supplementation did not influence muscle performance or response to exercise in healthy young individuals [216, 217] • No effect of NR supplementation on muscle mitochondrial content and respiratory capacity in overweight or obese pre-diabetic individuals [218, 210]
AGE-RELATED DECLINE (I): CARDIOVASCULAR FUNCTION	• NR improved cardiac NAD^+^levels, cardiac function and premature death in a mouse model of altered LMNA function [219] • NR attenuated the development of heart failure in models of dilated cardiomyopathy or transverse aorta constriction, as well as in response to pressure overload. [94, 220]	• NR reduced the levels of circulating pro-inflammatory cytokines in patients with Stage D heart failure [221] • No change in cardiovascular parameters, but reduced systemic inflammation in elderly [222] • NR supplementation reduced some parameters linked to hypertension [205]
AGE-RELATED DECLINE (II): MUSCLE FUNCTION	• NR reduced age-related protein deposits in skeletal muscle [223] • NR preserved muscle stem cell function in aged mice [76]	• NR did not improve muscle mitochondrial function or strength in old individuals [222] • Acute NR intake (2 h prior to exercise) increased performance in aged individuals [217]
AGE-RELATED DECLINE (III): OTHER ASPECTS	• NMN improved age-related insulin resistance [74]	• NR did not influence glucose tolerance in elder individuals [222]
OTHER INDICATIONS	• NR improved muscle performance in mouse models of mitochondrial myopathy [224] • NR extended lifespan in pre-clinical models of ataxia telangiecstasia (AT) [225]	• Nicotinic acid (NR not tested) improved muscle function in patients of mitochondrial myopathy [226] • NR improved several kinetic and speech parameters in patients of AT [227]

Pre-clinical interventions are limited to those in rodents and, unless otherwise stated, to those in mice

#### Nicotinamide riboside, obesity and metabolic disease

Experiments in mouse models revealed that, despite effectively increasing NAD^+^ levels in multiple tissues, NR had very weak effects on metabolic parameters in healthy young mice fed a normal diet, [152]. However, NR-treated mice were protected against diet-induced body weight gain and glucose intolerance [152]. These effects could be related to an increase in energy expenditure, which correlated with higher SIRT1 and SIRT3 activity—based on the decreased acetylation levels of target proteins—increased mitochondrial content and thermogenic capacity in BAT [152]. In relation to thermogenesis, an independent study confirmed that NR can influence thermogenic capacity, even in lean mice, after only 5 weeks of NR supplementation [228]. The ability of NR to improve glucose homeostasis was also confirmed in parallel studies were NR was administered to mice made glucose intolerant through either the combined action of high-fat diets and streptozotocin [84] or through high-fat high-sucrose diets [80]. These studies suggested that the liver seems to be the major target tissue for NR in the treatment and prevention of metabolic disease. This is logic, as the liver is one of the tissues expressing higher NRK1 levels [163]. In agreement, NR administration prevented non-alcoholic fatty liver disease (NAFLD) [80, 84], alcohol-related liver damage [89] and diet-induced hepatic fibrosis [212]. Strikingly, NR did not only prevent, but also reverted fatty liver disease in mice [80]. Other lines of experiments have also demonstrated that NR can enhance liver regeneration after partial hepatectomy [213]. The role of NR metabolism in protecting hepatic function was further supported by the observation that NRK1 liver-specific KO mice display exacerbated glucose intolerance and liver damage in response to HFD [85].

The protection of NR against diet-induced metabolic damage, however, has not been universally observed in the mouse studies carried out to this date. Recent works have reported either very marginal or no effects of NR on glucose metabolism, hepatic lipid accumulation or energy expenditure in diverse C57Bl/6 mouse strains submitted to HFD [206–208]. The reasons for these discrepancies are unclear but might be related to some important aspects beyond the small differences between the intervention designs. First, in the initial reports where NR protected against diet-induced obesity, NR was administered as a trifluoromethanesulfonate (triflate) salt, mixed with food at an approximate dose of 400 mg/kg/day [80, 152]. Triflate salts, however, are not considered as pharmaceutically acceptable for human use, which led to the development of the chloride salts that have been used in the most recent studies, where NR supplementation has often led to more modest [207] or no [206, 208] effects on glucose homeostasis at various doses. A second factor that could play a major role are differences in animal housing conditions. A recent analysis comparing age and diet-matched mice in four different animal housing centers with large experience and know-how in metabolic phenotyping, revealed large variance in the metabolic rate between the sites [229]. Consequently, the response of these mice to HFD was highly variable, with mice at one site gaining three times more fat mass than mice at another site [229]. This might have a significant impact on NAD^+^ turnover and NAD^+^ precursor requirements. Altogether, these observations indicate that the effects of NR on metabolic disease are variable and might largely depend on the degree of tissue-damage attained by HFD and the resulting NAD^+^ turnover at the organismal, tissular and cellular level. Additional pre-clinical studies will be needed to understand the variables influencing the therapeutic potential of NR in situations of metabolic disease.

In humans, two independent trials failed to detect an influence of NR on insulin sensitivity in obese, insulin resistant, but otherwise healthy individuals. First, in a randomized, placebo-controlled, double-blinded clinical trial, Dollerup and colleagues demonstrated that 12 weeks of NR supplementation (2 g/day) does not lead to improvements in insulin sensitivity or body composition [202]. Two follow up reports on the same clinical trial indicated that NR also did not influence the circulating levels of a collection of markers for pancreatic function [209], and had no impact on skeletal muscle mitochondrial content or respiratory capacity [218]. A piece of information that gathered a lot of attention from this intervention was that a 2% reduction in hepatic lipid content was observed in the NR supplemented group, compared with a 0.2% reduction in the placebo group. This difference, however, did not attain statistical significance. Given that hepatic lipid content was not the primary endpoint of the study, the clinical trial might not have been powered enough for this particular readout. Hence, it will be interesting to see the outcome of future clinical studies devoted to the evaluation of NR effects on hepatic lipid accumulation in fatty liver disease patients. In this sense, a clinical study using NR, in combination with other molecules (L-serine, L-carnitine tartrate and n-acetyl L-cysteine) for 70 days in NALFD patients suggests that this combination could reduce liver fat, as well as the circulating levels of alanine and aspartate aminotransferases [214]. The study design does not allow to conclude to what degree these benefits could be attributed to the effects of NR itself, and the overall effect on glucose homeostasis was not evaluated.

In an independent study, Remie and colleagues performed a randomized, double-blinded, placebo-controlled, cross-over intervention study on healthy overweight individuals, evaluating the influence of NR (6 weeks, 1 g/day) on multiple metabolic parameters [210]. In this study NR supplementation did not alter insulin sensitivity, skeletal muscle mitochondrial respiratory capacity or cardiovascular parameters. No effects of NR were seen on hepatic lipid accumulation, but this could be attributed to the fact that hepatic lipid content in the individuals from this trial were relatively low and within a healthy range. Nevertheless, some minor effects of NR were observed. First, NR promoted a slight decrease in total body fat mass, accompanied by an increase in lean mass [210], which could be related to a higher sleeping metabolic rate [210]. This clinical trial also reported an increased acylcarnitine formation in response to exercise in patients treated with NR [210]. Hence, NR might favor lipid mobilization under stress situations, but this hypothesis still requires further substantiation.

Both clinical trials reported that muscle mitochondrial protein content and respiratory capacity were not affected by NR supplementation [210, 218], which was also observed in a third clinical trial performed in elder individuals by Elhassan et al. [222]. The poor effects of NR on muscle metabolism might be explained by multiple factors. First, because NRK1 is poorly expressed in skeletal muscle, compared to liver [163]. Second, because the muscle has a very strong reliance on internal NAM salvage to maintain NAD^+^ levels [230, 231]. Third, because after intraperitoneal, intravenous or oral supplementation, NR is quickly degraded and poorly reaches muscle tissue [51, 163]. Finally, unlike liver, NRK1 deficiency in mice does not alter muscle mitochondrial respiratory capacity or weight, even under high-fat feeding ([85] and Audrey Sambeat, Joanna Ratajczak and Carles Canto, unpublished observations). Not surprisingly, NR failed to enhance exercise performance in healthy young humans [216, 217], even if slight improvements were observed in elder individuals [217].

#### Nicotinamide riboside and age-related diseases

NR has been shown to increase lifespan in yeast [232] and worms [70]. On average, worms lived 20% longer and the effect seemed to be NAD^+^ related, as other precursors reached similar effects [70]. Initial evidence suggested that this could also be the possibility in mice. When 24-month-old C57Bl/6JRj mice were supplemented with NR (400 mg/kg/day), they showed a ~ 4% longer lifespan [76]. However, a larger effort from the interventions testing program (ITP) using genetically heterogenous mice fed NR (1 g/kg of food) from 8 months of age failed to find any difference in the lifespan of male or female mice [233].

The fact that maximal lifespan might not be influenced by NR should not understate the relevance of NAD^+^ metabolism in age-related diseases. Several mouse interventions using NAD^+^ precursors have shown that they can mitigate age-associated physiological decline, either in mice fed regular diets [234] or HFDs [235]. In a study were 5-month old mice on a normal diet where supplemented with NMN for 12 months, NMN-treated (300 mg/kg/day) mice displayed lower age-associated body weight gain, increased energy expenditure and spontaneous physical activity, improved insulin sensitivity and lipid profiles as well as higher bone mineral density [234]. Unfortunately, the ITP study did not report intermediate data on glucose management or physical performance that could be cross compared with this study. However, there are several reasons to believe that we should expect similar outcomes from NMN and NR interventions. Most notably, genetic and tracer analyses support that NMN must be either dephosphorylated to NR or further cleaved to NAM in order to enter the cell and act as a NAD^+^ precursor [162, 163, 236, 237]. The most compelling proof in this direction is that the action of NMN as a NAD^+^ precursor is compromised in cultured cells and in mouse tissues upon NRK1 deletion [163]. This concept has been recently challenged by the identification of a potential NMN transporter, Slc12a8 [238]. This finding, however, has raised some controversy [239] and future studies will have to define the physiological role of Slc12a8 as a path for NMN-induced NAD^+^ synthesis vs. its conversion to NR or NAM.

Another avenue used to evaluate the therapeutic possibilities of NR in the prevention and treatment of age-related physiological decline has been the use of premature aging model organisms. Work from the Bohr lab has demonstrated that NR improves several features of mouse models (Csb^m/m^) for the Cockayne Syndrome (CS), a progressive neurodegenerative accelerated aging disorder caused by mutations in the CSA or CSB genes which encode proteins involved in DNA repair and transcriptional regulation [112, 240]. Csb^m/m^ mice display many features of mild human CS such as smaller brain weight, inner ear pathology, neuroinflammation and weight loss. One week of NR treatment was enough to normalize ATP levels, mitochondrial function and transcriptional signatures in the cerebellum of Cbs^m/m^ mice [112]. A short-term (10 days) treatment with NR also counteracted cochlear NAD^+^ deficits and prevented the sharp progression of hearing loss in CS mouse models [241]. Interestingly, NR also protected against noise-induced hearing loss in mice [109, 242], further supporting the ability of NR to prevent axonal degeneration in the inner ear.

The most well-known premature aging syndrome is humans is the Hutchinson–Gilford progeria syndrome (HGPS), an autosomal-dominant genetic disease caused by mutations in the LMNA gene, that leads to accelerated aging and often premature death caused by cardiovascular complications. Mouse models of HGPS do not fully recapitulate the human symptomatology of the disease [243], yet still provide useful information to understand the basis of the cardiovascular complications in these patients. Further, specific mutations in the *LMNA* gene can lead to cardiovascular complications in the absence of an overt progeroid syndrome [244]. In this sense, mouse models carrying a *LMNA*^*H222P*^ mutation develop overt cardiomyopathy at early stages of life, accompanied by decreased NAD^+^ levels in cardiac muscle [219]. Feeding *LMNA*^*H222P*^ mice with NR (400 mg/kg/day) not only recovered cardiac NAD^+^ levels, but also improved cardiac function and prevented their premature death [219]. Interestingly, *LMNA*^*H222P*^ mice displayed increased expression of *Nmrk2* in cardiac tissues [219]. Higher mRNA and protein levels of NRK2 are also observed in human and mouse heart samples of dilated cardiomyopathy and failing hearts [94, 182]. It is also interesting to note that the sole deletion of the *Nmrk2* gene in mice is enough to accelerate a decline in cardiac function in aged mice or in response to myocardial infarction [181, 182]. In line with this, NR supplementation attenuated the complications of multiple mouse models of heart failure, including dilated cardiomyopathy, transverse aorta constriction or pressure overload [94, 220]. Human patients with stage D heart failure orally supplemented with NR for 5–9 days (0.5–1 g/day) displayed increased whole blood NAD^+^ levels, mitochondrial function in peripheral blood mononuclear cells (PBMCs) and a large reduction in pro-inflammatory cytokine (IL-1b, IL-6 and IL-18) gene expression [221]. This suggests that NR could help reducing the pro-inflammatory systemic state in in cardiovascular complications. Along the same lines, when healthy elderly individuals where supplemented with NR (1 g/day) for 21 days no major changes were observed in body composition or cardiovascular parameters, yet a decrease in circulating levels of inflammatory cytokines (IL-2, IL-5, IL-6) was also observed [222]. Therefore, two independent studies point towards the concept that NR supplementation could reduce systemic inflammatory signals.

Aging is also associated with neurodegenerative diseases and cognitive decline. Initial evidence for the protection of NR against neurodegenerative disorders was obtained in the Tg2576 Alzheimer’s disease (AD) mouse model, which overexpresses a mutant form of the amyloid-beta (Ab) precursor protein with the KM670/671NL mutation, resulting in elevated levels of Aβ and ultimately amyloid plaques. This mouse model is characterized by increased age-related cognitive decline. The administration of NR (250 mg/kg/day, via drinking water) for 3 months significantly improved cognitive performance in an object recognition test [245]. This was accompanied by increased expression of mitochondrial-related genes in the brain and improved synaptic plasticity in NR-treated mice [245]. In another study, the effect of NR on Alzheimer’s disease was evaluated using the APP/PSEN1 AD mouse model. There, NR (400 mg/kg/day, mixed with food) robustly reduced Aβ deposits in the brain cortex tissues of AD mice and oxidative phosphorylation protein levels, culminating in increased context-dependent memory, as assessed by contextual fear conditioning tests [246]. NR supplementation also reduced age-related protein deposits in skeletal muscle [223]. The ability of NR to reduce protein aggregates might be related to triggering the mitochondrial unfolded protein response (mtUPR) in most tissues and organisms tested to date [70, 80, 224, 246], yet confirmation in humans is still missing.

Recently, NR showed some promising outcomes in treating patients suffering from ataxia–telangiectasia (AT), a rare neurodegenerative disease, causing severe disability. AT is caused by mutation of the Ataxia-Telangiectasia Mutated (ATM) gene, encoding the ATM kinase, a master regulator of DNA damage resolution [247]. Initial work in mice suggested that dietary NR supplementation (12 mM in the drinking water) increased the lifespan of the short-lived *Atm*^*−/−*^ mice, correlating with the normalization of mitochondrial architecture due to improved mitophagy and a better resolution of DNA repair [225]. In an open-label, proof-of-concept clinical study, 24 patients with AT were treated with NR (25 mg/kg/day) during four consecutive months. NR supplementation led to improvements in diverse kinetic and speech parameters [227]. Interestingly, these benefits disappeared 2 months after NR withdrawal [227], further strengthening that the benefits could be genuinely due to NR treatment.

## Lost in translation?

As the above section illustrates, the therapeutic effects of NR in the clinical setting remain rather modest. This might be surprising, given the successful therapeutic campaigns reported in most other organisms tested to this date. In this section we will discuss some aspects that are often overseen, but critically influence the interpretation of the results and the therapeutic potential of NR.

### NR degradation

We demonstrated that NR fails to increase NAD^+^ levels in primary hepatocytes from NRK1 KO mice [163]. Therefore, it came as a surprise when the action of intraperitoneally injected NR on hepatic NAD^+^ levels was only partially blunted in the absence of NRK1 [163]. Analysis of plasma metabolites one hour after NR injection revealed that NR levels were undetectable. However, a remarkable increase in circulating NAM was observed, which could explain the partial effects on NAD^+^ levels [163]. Prior work had described how NR could be degraded in the gut to NAM [143]. However, given the intraperitoneal nature of the compound delivery in our experiments, other mechanisms should account for the great rise of NAM after NR administration. Experiments in which NR was spiked into isolated mouse plasma revealed that NR can rapidly degrade to NAM [163]. This suggested that NR degrades in circulation and that the residual effects of NR on tissue NAD^+^ observed in the NRK1 KO mice derived from the production of NAM. Interestingly, very similar observations were obtained with NMN, which increased NAM in a comparable fashion to that of NR [163]. However, NMN did not degrade in isolated plasma, yet did so in the presence of cells, suggesting that NMN requires ecto-cellular enzymes to be converted to NR, which could include CD38 [73] or CD73 [236], amongst others [248].

While we knew that NR could be degraded to NAM in circulation, our experiments were not able to quantify how much of the injected NR reached mouse tissues prior to its degradation. Bringing light into this question, the Rabinowitz lab performed elegant tracer analyses coupled to flux quantification. Their results illustrated that when NR and NMN were administered by an intravenous (IV) bolus at 50 mg/kg, NR could be detected in circulation at 5 and 15 min after administration but became undetectable by 45 min [51]. NR could also be detected in circulation 15 min after NMN administration, yet NMN itself seemed virtually indetectable [51], in agreement with previous reports [163, 249]. When administered orally, no increases in NR or NMN were detected in circulation. Irrespective of the route of delivery, the main circulating product of the administered NR or NMN was NAM [51]. These observations certify that NR and NMN are largely degraded to NAM after systemic or oral administration. However, the agents responsible for this degradation have not yet been fully established. Recent reports suggest that the gut microbiome can account for a large portion of the transformation of oral NR [250, 251]. However, the fact that NR leads to NAM production in circulation after IP and IV administration, strongly suggest that a circulating protein might be able to cleave NR. A likely candidate is the purine nucleoside phosphorylase 1 (PNP1) enzyme. PNP1 has been shown to cleave NR to NAM intracellularly in eukaryote cells [232, 252], but PNP1 has also been detected in circulation [253, 254], which could facilitate NR transformation.

**Fig. 4:**
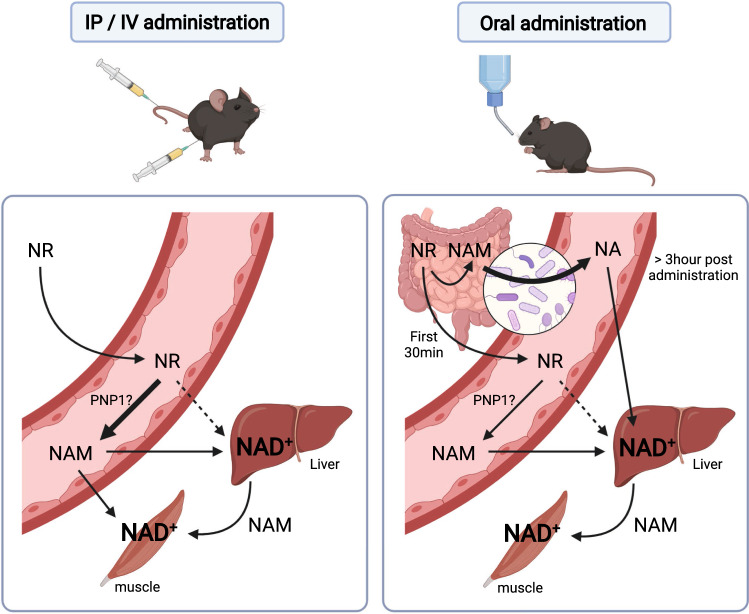
Distribution of NR after administration in mice. After intraperitoneal (IP) or intravenous (IV) administration, NR goes into the bloodstream, where it is partially degraded to NAM. NR is incorporated in several tissues, such as liver, but, at least after 1 h, most of the effect on peripheral tissues, such as muscle, can be attributed to NAM generated from NR degradation (left panel). After oral administration, NR is slowly degraded to NAM in the gut, where NAM is subsequently transformed to NA. During the first 30 min after oral intake, NR can be absorbed, following similar fates as those described for IP/IV administration. However, after 3 h, most of the sustained actions of NR on NAD^+^ metabolism might be attributed to NA generated in the gut

One implication of the high volatility of NR in circulation is that NR might poorly target tissues after IP, IV or oral administration. After IP injection part of the action of NR on NAD^+^ levels was blunted in NRK1 KO mice [163], indicating that NR is still reaching tissues in significant amounts to increase NAD^+^ levels. However, the influence of NRK1 deletion was tissue dependent. For example, NR action in liver and kidney was largely impaired in NRK1 KO mice, but this was not the case in muscle tissue, where NR increased NAD^+^ similarly in WT and NRK1/NRK2 double KO mice [163]. Tracer analyses provided a plausible explanation for this phenomenon, as after IV or oral administration, NR did not directly incorporate into NAD^+^ in muscle [51]. Instead, NR-driven NAD^+^ synthesis in muscle was largely driven by the NR degradation product, NAM [51] (Fig. 4).

The above observations are in line with the fact that NRK1 expression in skeletal muscle is relatively low [163], which suggests that NR might be a poor physiological modulator of NAD^+^ levels in this tissue. Instead, muscle tissue might strongly rely on NAM to sustain NAD^+^ levels. In line with this, two independently created muscle-specific NAMPT-KO mouse lines demonstrated that *Nampt* deletion in muscle leads to a ~ 85–90% depletion in muscle NAD^+^ content, which translated into decreased muscle strength, muscle fiber size and impaired mitochondrial respiratory capacity [230, 231]. In one of these mouse lines, these features led to death in early life stages [231]. Strikingly, NR could partially rescue some of these phenotypes and delayed muscle degeneration [230, 231]. According to tracer experiments, a minute amount of NR can reach muscle tissue in muscle-specific NAMPT-KO mice, even after oral administration [230]. The exact reason why NR incorporation in the NAD^+^ pool is manifested in the NAMPT KO mice but not in wild-type (WT) mice is not understood but could be consequent to different turnover and incorporation rates, especially when considering the differences in baseline NAD^+^ levels between WT and NAMPT KO micet. These results provide demonstration that direct utilization of NR by the muscle can occur after oral administration, albeit at very marginal levels.

A fascinating twist has been recently added to the NR story by suggesting that orally administered NR, as well as NMN or NAM, will be almost fully transformed to NA by the gut microbiome, which then accounts for the effects on NAD^+^ synthesis in mouse tissues [250]. Most shockingly, if the luminal conversion to NA was impeded, NR failed to increase hepatic NAD^+^ levels [250]. This was confirmed to a large degree in a second study, which suggested that NR could act in two phases. In a first phase (~ 30 min after oral delivery), NR could act as a NAD^+^ precursor without conversion to NA [251]. In a second phase (~ 3 h), the degradation of NR to NAM prompts its conversion to NA by the gut microbiome, which then serves as a sustained NAD^+^ precursor for, at least, the intestine and the liver [251] (Fig. 4). These works have provided essential understanding on the role of gut microbiome to whole-body metabolism and explain why metabolites from the deamidated NAD^+^ synthesis paths are increased upon NR administration [153]. However, they also raise some questions. First, if NR or NAM are largely transformed to NA, one would predict that the oral administration of these compounds should also cause flushing, which is experienced by most patients at NA doses of 0.5 g/day [134, 255]. Yet, neither NAM nor NR cause flushing in humans even at four–sixfold higher doses [202, 256]. One important point here could be that the gut microbiome can largely differ from mice to humans. A second conflicting element that deserves attention is that NAM and NR can induce NAD^+^ synthesis in cultured cells and in mouse tissues after IP or IV administration [51, 163], which makes it unclear why their transformation to NA in the gut was required to influence hepatic NAD^+^ after oral administration (Fig. 4).

Altogether, the above studies might explain why NR has failed to provide any major effect in skeletal muscle metabolism in the different clinical trials performed to this date on healthy obese or elder subjects [202, 210, 218, 222]. First, orally administered NR will poorly reach muscle tissue directly and, rather, will substantially resemble NAM or NA supplementation. Increasing NAD^+^ synthesis in muscle via the NAMPT pathway might have little or no consequences on muscle metabolism, as highlighted by muscle-specific NAMPT overexpressing mice [257, 258]. Alternatively, if NR is converted to NA by the gut microbiome, NA will poorly be utilized in muscle, as this tissue lacks NAPRT expression [141]. Finally, the experiments in NAMPT-KO mice suggest that the muscle tissue has a great reserve of NAD^+^ levels and, under most physiological conditions, NAD^+^ levels might be largely enough to maintain its essential functions.

### New horizons

NAD^+^ precursors are rarely compared side by side. Hence, it remains unclear to what degree they are redundant or have some unique actions. Several observations, however, argue for the later. First, the fact that NA can activate GPR109A or that NAM could lead to end-product inhibition of NAD^+^ consuming enzymes already sets a frame for specific actions of different NAD^+^ precursors. Second, some reports have observed differential effects of NR and NAM under the same experimental settings. For example, NR could rescue cardiac function in *LMNA* cardiomyopathy, but not NAM [219]. Also, NR and NMN showed effectiveness in expanding hematopoietic progenitors after bone marrow transplantation, but not NA or NAM [259]. Finally, the fact that NRK1 KO mice display phenotypes suggest that other precursors cannot fully support NAD^+^ synthesis in certain scenarios, such as high-fat feeding [85]. Importantly, supplementing NRK1 KO mice with NAM did not improve their exacerbated metabolic damage [203]. All the above elements indicate that NR could have unique therapeutic actions, compared to NA or NAM. However, NR is largely transformed to NAM and NA in the gut and circulation, which would curtail a large part of the potential advantages of using NR vs. classical NAD^+^ precursors.

The work with NAMPT KO mouse models has shown that orally administered NR could still be incorporated to NAD^+^ even in peripheral tissues, such as skeletal muscle [230]. Situations of largely impaired NAD^+^ levels might, hence, favor using the NRK path, which is in line with the increases in *Nmrk2* expression observed in in response to diverse challenges in mice (see Sect. 3.2.2). In this sense, it is important to note that despite most clinical trials indicated that NAD^+^ boosters poorly influence muscle metabolism, this was not the case in patients suffering from mitochondrial myopathy, where increasing NAD^+^ led to significant increases in muscle strength and mitochondrial biogenesis [226]. Therefore, it will be of critical importance to identify human conditions where the NAD^+^ impairments can determine physiological performance. In this sense, the fact that mice have a ~ sevenfold higher metabolic rate [260] might favor the generation of situations of high NAD^+^ demands upon physiological or pathological stresses, therefore magnifying the outcomes of NAD^+^ precursors supplementation compared to humans.

Given the unique characteristics of NR, one important goal would be to prevent NR degradation in the GI tract or in circulation. In this sense, several derivatives of NR have been generated by introducing various chemical groups on the backbone of the molecule [261, 262], but their influence on NR bioavailability is still to be determined. However, it is often overseen that even if NR degradation is prevented, NR action will be determined by hepatic first pass and by NRK activity, which is high in liver and kidney, but more modest in other tissues. In other words, even when maximizing NR levels in circulation, some tissues might remain poorly responsive due to limited NRK activity.

A curious finding might be able to bypass the above limitations. Parallel work by the Sauve lab and our group identified a reduced version of NR, dihydronicotinamide riboside (NRH), as a new NAD^+^ precursor in mammalian cells and tissues [128, 263] (Fig. 2). Despite the structural similarity with NR, the biological properties of NRH turned out to be particularly surprising. NRH can sharply increase NAD^+^ levels in cultured cells, being far more potent than any other NAD^+^ precursor described to date [128, 263]. After entering the cells, predominantly through ENTs [128], NRH uses a unique path to drive NAD^+^ synthesis. Surprisingly, the ability of NRH to act as a NAD^+^ precursor does not rely on NRK activity. Using chemical inhibitors, Giroud-Gerbetant et al. identified adenosine kinase (AK) as the enzyme that initiated the conversion of NRH to NAD^+^ [128]. This finding was later confirmed by the Sauve lab using cellular fractionation methods [264]. The phosphorylation of NRH by AK renders dihydronicotinamide mononucleotide (NMNH), which is then adenylated by NMNAT enzymes to generate NADH, which is then oxidized to NAD^+^ [128, 264]. Thus, NRH defines a new path towards NAD^+^ synthesis relying on the activity of AK. The use of AK for the initial catalysis step and that it acts by increasing NADH might be critical in understanding why NRH action in cultured cells is so vastly superior to all other precursors. Interestingly, as with NMN, NMNH can be used as an effective extracellular NAD^+^ precursor, but also require dephosphorylation to NRH prior to cellular uptake [265].

Another interesting aspect of NRH is that, unlike NR, it is not degraded in mouse plasma [128]. Accordingly, the intraperitoneal injection of NRH led to larger increases in NAD^+^ than those observed with NR [128]. Unlike NR, NRH could be detected in circulation after oral administration. More precisely, it was detected in the low micromolar range when gavaged at 250 mg/kg, which is consistent with promoting significant effects on NAD^+^ levels in cultured cells [128]. To date, two studies in mice support the case that NRH could have therapeutic applications. First, NRH administration (250 mg/kg/day) protected against cisplatin-induced acute kidney injury (AKI) [128]. NRH counteracted cisplatin-induced decreases in NAD^+^ levels. In turn, this allowed maintaining PARP activity and preventing the formation of kidney casts [128]. In a second study, NRH displayed protection against ethanol-induced hepatic toxicity. The oral administration of NRH (500 mg/kg) 15 min prior to an acute bolus of alcohol led to accelerated first pass ethanol metabolism, reducing circulating alcohol levels and prompting its ultimate transformation to acetate. This, in turn allowed NRH to prevent alcohol-induced hepatic damage, based on alanine and aspartate aminotransferase (ALT and AST, respectively) measurements.

In contrast to other NAD^+^ precursors, however, NRH can manifest cellular toxicity. HepG3 cells treated with NRH showed some signs of cellular toxicity, related to an increase in mitochondrial superoxide production [266]. Interestingly, these effects were cell-specific, as NRH did not prompt toxic effects in HEK293 [266]. Nevertheless, this could be in line with the increased PARylation that has been observed in response to NRH treatment in cultured cells as well as in mouse liver and kidney tissues [128, 264, 266, 267].

## Conclusions and future directions

NR was made available in 2013 as a supplement in a form of crystalline chloride salt, under the brand name NIAGEN (Chromadex Inc., Irvine, CA, USA). This development led to numerous trans-sectorial collaborations evaluating the potential therapeutic benefits of NR in mammalian organisms. A second game-changing development in the field has been the synthesis of labeled NR and NAD^+^-related compounds that have allowed the deployment of tracer studies.

If anything, the last decade of research in the NAD^+^ field has highlighted how our previous understanding of NAD^+^ biology was far from complete. The discovery of NRH has clearly illustrated that the NAD^+^ universe might expand far beyond the boundaries established in the early 2000s. Further, we might have overseen how multiple NAD^+^-related molecules might enzymatically and non-enzymatically react and transform before reaching its target tissues. The experiments in animal models have illustrated that different NAD^+^ precursors are unique, but also that interorgan communication plays a critical role in whole-body NAD^+^ homeostasis, an aspect that can be poorly mimicked using cultured cell studies. Finally, pre-clinical interventions on NR and other NAD^+^ precursors, have demonstrated a wide range of variability in their outcomes, either due to different animal housing conditions or the recently discovered impact of the gut microbiome on whole-body NAD^+^ regulation. All the above points constitute a very valuable advance in our knowledge, but also underscore the multiple complications surrounding the clinical translation of NAD^+^-based therapeutics.

Future pre-clinical and clinical studies are needed to evaluate and validate the therapeutic potential of NR and other NAD^+^ precursors. New studies will benefit from, whenever possible, comparing the effectiveness of different NAD^+^ precursors side by side. We also need a better understanding of human NAD^+^ metabolism and in which situations therapeutic supplementation might be necessary. In this sense, it would be worth exploring which NAD^+^ metabolites could act as readouts to identify pathophysiological situations requiring NAD^+^ precursor supplementation in humans. Finally, pre-clinical efforts will still be required to understand the mechanisms of action for NAD^+^-related benefits in different disease settings.

The clinical translation of pre-clinically successful molecules is most often a bumpy road, and this is being no exception for NR. Nevertheless, most data indicates that NR plays unique and differentiated roles compared to other NAD^+^ precursors. Innovations on delivery formats, chemical protection or other methods to increase its bioavailability will be needed to unlock its full potential.

## Data Availability

Not applicable.
